# Effect of Artemisinin-Loaded Mesoporous Cerium-Doped Calcium Silicate Nanopowder on Cell Proliferation of Human Periodontal Ligament Fibroblasts

**DOI:** 10.3390/nano11092189

**Published:** 2021-08-26

**Authors:** Ioannis Tsamesidis, Dimitrios Gkiliopoulos, Georgia K. Pouroutzidou, Evgenia Lymperaki, Chrysanthi Papoulia, Karine Reybier, Pierre Perio, Konstantinos M. Paraskevopoulos, Eleana Kontonasaki, Anna Theocharidou

**Affiliations:** 1 Department of Prosthodontics, School of Dentistry, Faculty of Health Sciences, Aristotle University of Thessaloniki, GR-54124 Thessaloniki, Greece; itsamesidis@auth.gr (I.T.); antheo@dent.auth.gr (A.T.); 2 Laboratory of Advanced Materials and Devices (AMDeLab), School of Physics, Faculty of Sciences, Aristotle University of Thessaloniki, GR-54124 Thessaloniki, Greece; gpourout@physics.auth.gr (G.K.P.); cpapouli@physics.auth.gr (C.P.); kpar@auth.gr (K.M.P.); 3 Laboratory of Chemical and Environmental Technology, School of Chemistry, Aristotle University of Thessaloniki, GR-54124 Thessaloniki, Greece; dgiliopo@chem.auth.gr; 4 Department of Biomedical Sciences, International Hellenic University, GR-57001 Nea Moudania, Greece; evlimper@gmail.com; 5 PharmaDev, UMR 152, Université de Toulouse, IRD, UPS, 31000 Toulouse, France; karine.reybier-vuattoux@univ-tlse3.fr (K.R.); pierre.perio@univ-tlse3.fr (P.P.)

**Keywords:** mesoporous nanopowders, cerium doping, artemisinin loading, hemocompatibility, human periodontal ligament fibroblasts

## Abstract

Ion doping has rendered mesoporous structures important materials in the field of tissue engineering, as apart from drug carriers, they can additionally serve as regenerative materials. The purpose of the present study was the synthesis, characterization and evaluation of the effect of artemisinin (ART)-loaded cerium-doped mesoporous calcium silicate nanopowders (NPs) on the hemocompatibility and cell proliferation of human periodontal ligament fibroblasts (hPDLFs). Mesoporous NPs were synthesized in a basic environment via a surfactant assisted cooperative self-assembly process and were characterized using Scanning Electron Microscopy (SEM), X-ray Fluorescence Spectroscopy (XRF), Fourier Transform Infrared Spectroscopy (FT-IR), X-ray Diffraction Analysis (XRD) and N2 Porosimetry. The loading capacity of NPs was evaluated using Ultrahigh Performance Liquid Chromatography/High resolution Mass Spectrometry (UHPLC/HRMS). Their biocompatibility was evaluated with the MTT assay, and the analysis of reactive oxygen species was performed using the cell-permeable ROS-sensitive probe 2′,7′-dichlorodihydrofluorescein diacetate (H2DCFDA). The synthesized NPs presented a mesoporous structure with a surface area ranging from 1312 m^2^/g for undoped silica to 495 m^2^/g for the Ce-doped NPs, excellent bioactivity after a 1-day immersion in c-SBF, hemocompatibility and a high loading capacity (around 80%). They presented ROS scavenging properties, and both the unloaded and ART-loaded NPs significantly promoted cell proliferation even at high concentrations of NPs (125 μg/mL). The ART-loaded Ce-doped NPs with the highest amount of cerium slightly restricted cell proliferation after 7 days of culture, but the difference was not significant compared with the control untreated cells.

## 1. Introduction

Mesoporous materials have attracted considerable interest in the field of medicine. They present distinct advantages such as large surface area and tunable pore structure that render them ideal carriers for drug delivery, especially in combination with biologically active ions (e.g., Ca, Ce, Mg, Cu ions) [1,2]. In recent years, ion doping has appeared as a common strategy to improve the properties of mesoporous nanostructured biomaterials [3]. Bioactive mesoporous nanoparticles, bioactive glasses, nanopowders and scaffolds have been developed by the incorporation of ions such as calcium, magnesium, strontium, zinc, etc.

Calcium-doped mesoporous materials exhibit improved properties such as a stable mesh structure, high surface area and high bioactivity [4,5,6]. Both calcium and silicate ions can promote bone regeneration and increase bone mineral density in vivo [7,8,9], while a few studies have unraveled the effectiveness of bioactive mesoporous calcium-silicate nanoparticles on the apical sealing of teeth root canals [10] and the odontogenic differentiation of human dental pulp stem cells [11]. Magnesium and strontium doped calcium silicates have shown promising properties as drug carriers or as bone regenerative materials [12,13,14,15]. Recently, cerium ions (Ce^3+^, Ce^4+^) have attracted great interest as they attain multiple desirable characteristics, such as antioxidant [16], anti-inflammatory [17] and antimicrobial [18,19] properties, which are considered as prerequisites for tissue regeneration [20]. In a recent study, the incorporation of these ions into silicon-based mesoporous nanoparticles through a microemulsion assisted sol-gel method resulted in bioactive materials with antibacterial properties against *S. aureus* and *E. coli* [21]. It has already been argued that the addition of cerium (Ce) to mesoporous nanoparticles does not impair bioactivity while promoting cell proliferation [22].

Recently, a co-delivery concept has emerged where multi-functional materials with the capacity to simultaneously deliver different ions along with bioactive molecules, such as drugs or growth factors, are used for a synergistic combination of therapeutic effects on osteogenesis, cementogenesis or angiogenesis. Various ions (calcium, phosphorus, magnesium, strontium, cobalt, copper, cerium, boron, etc.), drugs and antibacterial/antimicrobial substances (dexamethasone, gentamycin, ibuprofen, ciprofloxacin, moxifloxacin, tetracycline, chlorhexidine, lysozyme, etc.), as well as growth factors (e.g., vascular endothelial growth factor (VEGF) and bone morphogenetic protein (BMP)) have been incorporated into MBGs with a high loading efficiency and effective release [3,23,24] along with regenerative potential [8]. Regarding the loading of mesoporous nanoparticles with proteins that constitute biomacromolecules with a large mass (i.e., rh-BMP2 26 kDa, BSA 66 kDa), pore size is one of the most critical factors affecting their adsorption within the mesoporous structure. The reaction conditions during synthesis (pH, temperature, surfactant type, etc.) play a significant role on the final pore size, while various pore size expanding agents have been tested to secure the intra-pore loading of proteins [25]. Mesoporous materials with large pore sizes and pore volume (>4 nm), such as the SBA-15 type, are efficient in incorporating large quantities of rhBMP-2 within their mesopores, also providing a sustained release of the protein [26]. On the other hand, the smaller pore sizes of the MCM-41 type, which are typically around 2–3 nm, are suitable for smaller drugs and antimicrobial substances.

Various mesoporous structures have been developed for the delivery of ions and drugs with applications in dentistry. Ambrogi et al. [27] loaded chlorhexidine on functionalized mesoporous MCM-41 nanoparticles and prepared poly(methylmethacrylate) based composites with Candida antibiofilm activity, while Yan et al. [28] used chlorhexidine-encapsulated mesoporous silica as a modifier of a commercial dental adhesive. Lu et al. [29] reported that the dual-controlled release of chlorhexidine and silver ions from biodegradable mesoporous silica nanoparticles was effective against S. mutans, suggesting their potential use as nanoantiseptics for oral biofilm therapies. In different applications, mesoporous bioactive glass nanoparticles containing SiO_2_, CaO and P_2_O_5_ have shown odontogenic and dentin regenerative potential in vitro, suggesting their use for the pulp-dentin complex regeneration [30], while bioactive silica-based MSNs doped with Ca, Mg and Sr can significantly increase human periodontal ligament fibroblasts’ (hPDLFs) proliferation, and could be used for periodontal ligament regeneration strategies [31]. Apart from mesoporous nanomaterials, the development of bioactive nanostructured bioceramics that combine mesoporous textural features with an apatite forming ability is a promising strategy for the local administration of substances to treat different pathologies of bony tissue such as infection, osteoporosis and even regeneration [32,33,34].

The function of Artemisinin (ART), an extract of Artemisia Annua, has been intensively studied since 1970, when it was discovered. Nowadays, it is considered by the World Health Organization (WHO) as the only potent drug to combat Malaria in combination with quinoline derivatives (Artemisinin based combination therapies). Recently, researchers demonstrated the beneficial impact of artemisinin in various biochemical pathways in different dental cells such as dental pulp stem cells (DPSC), bone marrow-derived mesenchymal stem cells (BMSCs) and human mesenchymal stem cells (HMSCs) [35,36]. Hu et al. [37] presented that artemisinin was able to restore the osteogenic differentiation of DPSCs under hypoxia conditions. Furthermore, the upregulated expression of CA9 and CA9-mediated antioxidant responses seemed to be the key process for the beneficial effect of artemisinin and the protective role of ART on DPSC osteogenesis. Fang et al. [38] observed the improvement of the in vitro survival of BMSCs after incubation with ART and especially when exposed to an ROS-induced environment, suggesting that artemisinin-mediated protection in BMSCs occurs via the activation of the c-Raf-Erk1/2-p90rsk-CREB signaling pathway. Zhang et al. [35] demonstrated that artemisinin compounds have the ability to inhibit osteoclast differentiation using their intracellular iron, activating the cleavage of Endoperoxide Bridge, generating ROS and causing oxidative damage and ferroptosis. In light of these results, we propose the loading of synthesized mesoporous nanocarriers with ART to verify their potential capability to locally deliver this multifunctional drug. Consequently, the aim of the present study was the synthesis, characterization and evaluation of the effect of artemisinin-loaded cerium-doped mesoporous calcium silicate nanostructured powders on hemocompatibility and the cell proliferation of human periodontal ligament fibroblasts.

## 2. Materials and Methods

### 2.1. Synthesis of Mesoporous Ce-Doped Nanopowders (Ce-NPs)

Mesoporous silica (Si-NP), calcium-doped (SiCa-NP) and calcium/cerium-doped silicate nanopowders (SiCaCe-NPs) were synthesized in basic environment (final solution with pH between 12 and 12.5) via a surfactant-assisted cooperative self-assembly process. For the synthesis of Si-NP, CTAB was initially dissolved in 800 mL of aqueous NaOH at 80 °C, forming a homogenous solution (Solution A). Another solution (Solution B) was prepared by dissolving TEOS in 200 mL of deionized H_2_O at room temperature under mechanical stirring. Next, Solution B was added to A dropwise and the mixture was left under stirring at 80 °C. After 2 h, the stirring was stopped, and the mixture was thermally aged at 100 °C for 24 h. The synthesized material was separated by filtration, washed once with ethanol and three times with deionized water, and was left to dry in atmosphere for three days. The organic phase (CTAB) was removed by calcination at 550 °C, for 6 h, in oxidative atmosphere, with a heating rate of 1 °C·min^−1^.

SiCa-NP and SiCaCe-NP were synthesized following the same procedure as the Si-NP. The only extra step was the addition of Ca and Ce nitrates in Solution B after the dissolving of TEOS and prior to the merging with Solution A. After the nitrate salts addition, Solution B was mechanically stirred until the salts were fully dissociated. The reactants molar ratios for the produced nanopowders are shown in Table 1 below.

### 2.2. Characterization of MSNs

#### 2.2.1. Scanning Electron Microscopy (SEM)

The morphology of the synthesized nanopowders was analyzed using Field-emission Scanning electron microscopy, JEOL JSM-7610F Plus supported by an Oxford AZTEC ENERGY ADVANCED X-act energy dispersive X-ray spectroscopy (EDS) system (JEOL Ltd., Tokyo, Japan).

#### 2.2.2. X-ray Fluorescence Spectroscopy (XRF)

Bulk analysis of the specimens was determined using a Bruker S4-Pioneer (Bruker AXS GmbH, Karlsruhe, Germany) XRF wavelength dispersive spectrometer equipped with an Rh tube, with the following five analyzing crystals: LIF200, LIF220, LIF420, XS-55 and PET. The detectors were a scintillation detector or a gas-flow proportional counter, or a combination of the two. Samples were analyzed at 60 kV and 45 mA tube-operating conditions. Specimens were prepared as glass beads by the fusion process using lithium tetraborate (LiT or Li_2_B_4_O_7_) as a flux. The ratio of specimen/flux was 1/8. The mixture was fused in a platinum crucible in a Vulcan fusion machine (Fluxana, Bedburg-Hau, Germany).

#### 2.2.3. Fourier Transform Infrared Spectroscopy (FT-IR)

Fourier transform infrared spectroscopy (FTIR) was used for the characterization of synthesized materials. The FTIR technique has been used to identify compositions of materials. FT-IR measurements of the synthesized materials were performed using a Spectrum 1000 (Perkin-Elmer, Waltham, MA, USA) spectrometer. Prior to measurement, the samples were grinded along with KBr (MSN:KBr ratio 1:100) and the mixture was molded into tablets using a hydraulic press. The spectra were collected after 10 scans in the range of 450–4000 cm^−1^.

#### 2.2.4. X-ray Diffraction Analysis (XRD)

To determine crystal structure and identify different crystal phases, X-ray powder diffraction (XRD) was performed. Small and Wide-Angle X-ray Analysis was performed using a Miniflex II XRD (Rigaku Co., Tokyo, Japan) diffractometer, with Bragg-Brentano θ–2θ geometry and Cu Ka radiation (λCuKa = 0.15405 nm). The scanning range was 1.5–10°, with 0.02° step size and 3.6-second step time and 5–85°, with 0.02° step size and 1-second step time, for Small and Wide-Angle X-ray Diffraction, respectively.

#### 2.2.5. N_2_ Porosimetry

The textural properties of the NPs were determined using porosimetry. Nitrogen adsorption/desorption experiments were performed at −196 °C to determine the specific surface area (multi-point BET method), total pore volume (at P/P0 = 0.99) and pore size distribution (BJH method using the adsorption data) of the samples, which were previously outgassed at 120 or 150 °C (hybrid and calcined silicas, respectively) for 16 h under 6.6 × 10^−9^ mbar vacuum using an Automatic Volumetric Sorption Analyzer (Autosorb-1, Quantachrome).

#### 2.2.6. Apatite Forming Ability

The synthesized materials were immersed in a simulate body fluid solution (c-SBF) with a concentration of 1.5 mg/mL and were maintained at 37 °C for 1 day. The c-SBF solution contains ion concentrations that are almost equal to those of the human blood plasma (Table 2) in pH of 7.30 to 7.40 [39]. The c-SBF was replaced 6 h after initial immersion. After 1 day, the samples were collected and left to dry at room temperature before FTIR analyses.

### 2.3. Preparation of ART-Loaded NPs and UHPLC/HRMS Analysis of ART Concentration

ART (1 mM) was added into 10 mL of alkynylated mesoporous NPs (1 mg/mL) solution, and the mixture was stirred (300 rpm) at room temperature in dark for 24 h. The ART-loaded NPs were separated from the suspension by centrifugation at 5000× *g* for 15 min and were then dried at room temperature. Each supernatant, after being properly diluted, was analyzed onto a UHPLC Kinetex EVO C18 1.7 µm, 2.1 × 100 mm column (Phenomenex, France) using UHPLC/HRMS system. The column temperature was set to 40 °C and the flow rate to 400 µL/min. The eluates used were H_2_O + 0.1% formic acid and ACN + 0.1% formic acid, using the following chromatographic conditions: 75% A/25% B to 95% B in 7 min, hold 1 min, back to initial conditions in 0.1 min and final hold until 10 min. The LTQ-Orbitrap XL ETD mass spectrometer (ThermoFisher Scientific, Paris, France) in positive electrospray ionization (ESI) mode. The voltage spray was set to 4.2 kV and the capillary temperature to 300 °C. The mass spectrometer to FTMS SIM scan type and the resolution to 15,000. The scan range was selected is 258–308 Da over the gradient time. The quantification of ART was based on a calibration curve previously prepared at 10, 100, 250, 500 and 1000 μM of ART. The loading capacity (LC) was calculated using the following equation: Loading capacity = [(Total amount of drugFree amount of drug)/nanoparticles weight] × 100. For the drug release studies, supernatants at different time points (0, 6, 17, 21, 24, 41, 48, 72, 96 h) were collected and stored appropriately and the artemisinin amount was quantified as previously described. In each experiment, the samples were analyzed in triplicate.

### 2.4. Biological Evaluation of MSNs

#### 2.4.1. Blood Sample Collection

Freshly drawn blood samples from healthy donors (both sexes were used) were collected in EDTA-tubes. All the donors provided informed consent to participate in the study. The study was conducted in accordance with Good Clinical Practice guidelines and the Declaration of Helsinki and was approved by Etablissement Français du Sang (EFS, Toulouse, France), responsible for ethic statements. Red blood cells (RBCs) were separated from plasma and leukocytes by washing three times with Phosphate buffer saline (PBS).

#### 2.4.2. Hemocompatibility Assay

To determine the hemocompatibility of RBCs with the NPs suspension (stock solution: 2 mg/mL), diluted RBCs were prepared in PBS in a final suspension consisting of 5% volume erythrocyte (final volume: 1 mL) (hematocrit: 5%). Diluted RBCs were treated with different concentrations of NPs (12.5, 30, 60, 125, 500, 1000 μg/mL) for 3, 15, 45, 60 min and 24 h of incubation at 37 °C (Thermomixer-Biosan). The supernatant of untreated RBCs was used as negative control (Ctrl-) and RBCs treated with lysis buffer were used as the positive control. All the samples were centrifuged at 2000 rpm for 1 min and a microplate reader (Thermo Scientific, Waltham, MA, USA) was used to measure the absorbance of hemoglobin release in the supernatant of treated samples. The absorbance value of hemoglobin at 541 nm was measured with the reference wavelength of 700 nm. The percent of hemolysis was calculated as follows:

(1) $$\mathrm{Hemolysis}\;\%=\frac{\;[\left(\mathrm{sample}\;\mathrm{absorbance}-\mathrm{negative}\;\mathrm{control}\right)}{\left(\mathrm{positive}\;\mathrm{control}-\mathrm{negative}\;\mathrm{control}\right)]}\times 100\%$$

Statistical analysis was performed with paired sample *t*-test. The level of statistical significance was set at 0.05.

#### 2.4.3. Isolation of Human Periodontal Ligament Fibroblasts (hPDLFs)

Human periodontal ligament fibroblasts cultures were established from human biopsies of periodontal ligament tissues of a healthy donor taken during routine third molar extraction. Small pieces of tissues produced by mincing were transferred into tissue culture flasks with 5 mL of DMEM supplemented with 10% fetal bovine serum (FBS, Invitrogen) and antibiotics (100 U/mL medium of penicillin, 100 mg/mL streptomycin, Invitrogen). The cultures produced were kept at 37 °C in an incubator in an air atmosphere with 95% humidity and 5% CO_2_. When a substantial fibroblast outgrowth (80% confluence) had been obtained, the cells were detached by trypsinization (using 0.25% trypsin/1 mM EDTA) and then subcultured under standard conditions and seeded in 24-well plates. The study was approved by the Institutional Ethical Committee (#110/10-2-2021).

#### 2.4.4. Cytotoxicity Assay

Cytotoxicity evaluation of NPs was performed using MTT. Cells were seeded in 96-well plates (1 × 10^4^ cells/well) and left for 24 h to attach at an incubator at 37 °C in a 5% CO_2_. Then, the cells were exposed to different concentrations of NPs. All the NPs (stock solution: 2 mg/mL) were disinfected with UV light for 90 minutes. A series of dilutions (12.5, 60 and 125 μg/mL) of NPs in the medium were added to the plate in sextuplicate. The cells were incubated with the NPs for 24, 72, 120 and 168 h at 37 °C in a humidified 5% CO_2_ atmosphere. Cells cultured with DMEM without FBS served as negative controls, while untreated cells cultured with complete medium served as positive controls. Two control groups and 5 NPs groups (each one tested at 3 different dilutions) were subjected to the MTT assay for cell viability determination. Six samples of each group were evaluated, and the experiments were performed in triplicate. Evaluation of mitochondrial activity and, thus, cell proliferation was calculated by measuring the mitochondrial dehydrogenase activity of living cells, which was verified by the transformation of the yellow tetrazolium salt into blue formazan crystals. DMSO was used as dissolvent. Optical density was determined using spectrophotometry at a wavelength of 545 nm and a reference filter of 630 nm using a microplate reader (Epoch, Biotek, Biotek instruments, Inc, Winooski, VT, USA). MTT assay values were presented as an average % percentage of the positive controls’ values. Statistical analysis was performed with paired sample *t*-test. The level of statistical significance was set at 0.05.

#### 2.4.5. Analysis of ROS Levels

The analysis of reactive oxygen species was performed using the cell-permeable ROS-sensitive probe 2′,7′-dichlorodihydrofluorescein diacetate (H2DCFDA) fluorescing at 520 nm (λex480 nm) upon oxidation. H2DCFDA probe (0.5 mM stock solution in DMSO) (incubated for 30 min) was incubated in the (1 × 10^4^ cells/well) treated with NPs at the same concentrations and time points as for the cytotoxicity assay. The monitoring of the measurement of the fluorescence of the desired suspensions in 96-well black microplates was performed using a SAFAS Xenius (Safas, Société Anonyme de Fabrication d’Appareillages Scientifiques, Monaco) fluorometer.

## 3. Results and Discussion

### 3.1. Characterization of MSNs

#### 3.1.1. Scanning Electron Microscopy (SEM)

**Figure 1:**
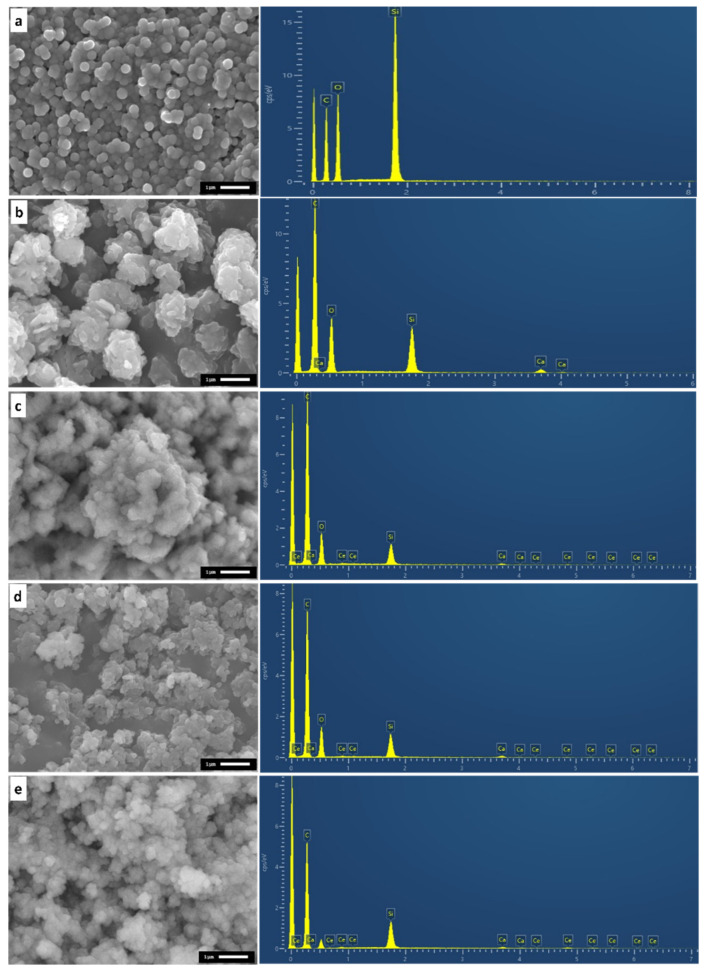
Representative SEM micrographs of the synthesized mesoporous nanopowders. (**a**) Si-NP, (**b**) SiCa-NP, (**c**) SiCaCe1-NP, (**d**) SiCaCe2.5-NP, (**e**) SiCaCe5-NP.

Representative SEM micrographs are presented in Figure 1.

As can be observed in Figure 1a, rounded shaped nanoparticles constitute the Si-NP nanopowder, while all the rest of the Ca- and Ce-doped nanopowders consist of aggregated nanoparticles of non-uniform shape. Sol-gel-derived mesoporous nanoparticles present a high reactivity and strong aggregation that is affected by multiple factors such as the surfactant content, drying process and presence of humidity [40,41,42]. Different strategies have been employed in the process of controlling aggregation [43]. These include the use of non-ionic co-surfactants that cover the reactive nanoparticles surface or other surface protecting agents, such as polyethylene glycol and amino acid L-lysine [44,45,46,47]. In the present study, the drying process was performed in atmosphere for three days, after washing with deionized water, and no other surface agent was applied to reduce the agglomeration. Furthermore, calcination at 550 °C to remove Ca and Ce nitrate salts may have assisted this aggregation tendency [48].

#### 3.1.2. X-ray Fluorescence Spectroscopy (XRF)

The chemical composition of all the synthesized NPs as detected using XRF is presented in Table 3. By comparing the nominal and the detected %wt. amounts, a slightly limited incorporation of CaO was observed in the NPs with CeO_2_, while the highest amount of CaO was calculated for the SiCa-NP. The actual composition of the NPs presented slight differences compared to the anticipated composition. Cerium doping resulted in a limited incorporation of both CaO and CeO_2_ in the NPs, as shown from the XRF analysis. This was expected, as numerous studies [1,49] have shown that in these systems calcium amounts are usually lower due to the co-doping of other ions. However, the amounts of both calcium and cerium were higher compared to those reported by Kurtuldu et al. [1] in the same system, and this explains the high bioactivity of the synthesized NPs, even those with the highest amount of cerium incorporation, that developed apatite after 1 day of immersion in SBF (see below). In the present study, a second, neutral medium to dissolve the nitrate salts and TEOS was used to minimize the formation of Ca(OH)_2_ or calcium and cerium oxides as byproducts. By the time the two solutions were merged, the formation of the silicate framework began instantly and there was a greater concentration of calcium and cerium cations available to be integrated. Furthermore, during the step of the hydrothermal ageing of the materials at 100 °C for 24 h, the polymerization of the silicate framework continued, and the wall thickness of the primary silica particles increased. It is possible that this enlargement also contributed to the increase in the calcium and cerium concentration.

#### 3.1.3. Fourier Transform Infrared Spectroscopy (FT-IR)

**Figure 2:**
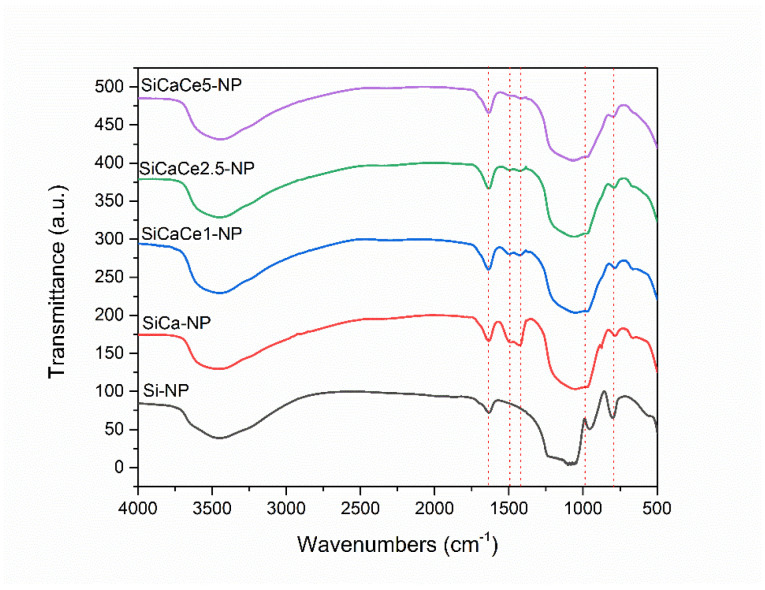
FT-IR spectra of NPs.

FT-IR spectra of NPs are shown in Figure 2. The broad band between 2800 and 3700 cm^−1^ is correlated to hydroxyl groups of absorbed water. The presence of absorbed water is also confirmed by the peak at 1635 cm^−1^, which corresponds to hydrogen bond vibrations between Si–OH groups and water molecules. The peak at 956 cm^−1^ also corresponds to the stretching vibrations of the Si–OH bond. This peak disappears in the Ca-containing samples and is replaced by a “shoulder” that widens the peak of 1000 cm^−1^. This behavior is attributed to the vibrations of the created Si–O–Ca bonds. The wide peak between 1000 and 1200 cm^−1^ corresponds to the asymmetric stretching vibrations of the Si–O–Si group bonds, while the peak at 800 cm^−1^ is attributed to the bending vibrations of the Si–O–Si group bonds. Finally, the peaks observed between 1410 and 1510 cm^−1^ in the Ca-containing samples are attributed to the vibrations of the C–O bond, that is correlated to carbonate species (CO_3_^2−^) physiosorbed from the air and the formation of CaCO_3_ [50]. The reduced intensity of these peaks at Ce-containing samples (SiCaCe), compared to the samples containing only Ca (SiCa), can be attributed to the incorporation of a lower number of calcium ions into the silicate lattice (phase out) due to the presence of cerium ions having an electrical charge of 3^+^ instead of 2^+^. Silica NPs form an open network structure of SiO_x_ tetrahedrons, which enables the incorporation of cations (such as Ca^2+^). These ions act as network modifiers, breaking the Si–O–Si bonds (bridging oxygens) and forming non-bridging oxygen groups (Si–O–NBO) that disrupt the glassy network [51]. The addition of the Ca and Ce ions led to the reduction in the peak around 800 cm^−1^, which is attributed to the Si–O symmetric stretching vibration. This decrease is related to the disrupted silicate network and indicates the incorporation of the ions into the glass network [52,53]. The incorporation of Ca led to stronger network connectivity compared to Si-NP, indicated by the decrease in the shoulder around 960 cm^−1^, while the addition of Ce led to a further reduction in the shoulder. Thus, it is concluded that Ca and Ce behave as network modifiers [31].

#### 3.1.4. X-ray Diffraction Analysis

**Figure 3:**
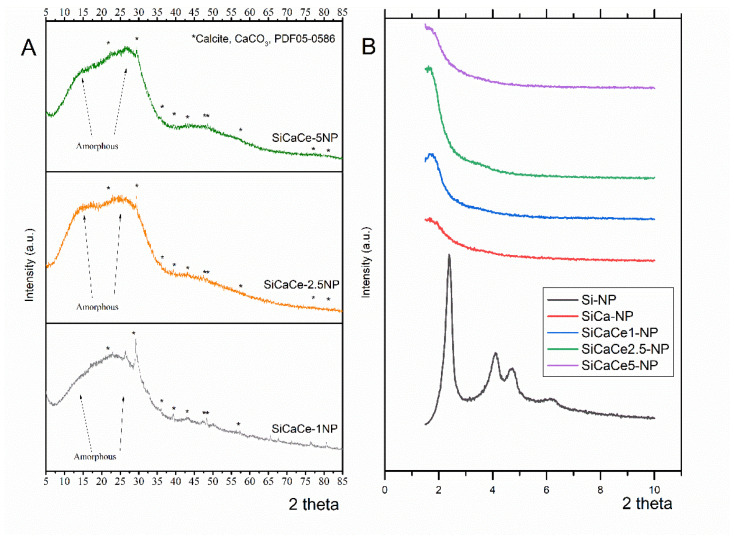
Wide- (**A**) and small-angle (**B**) XRD of NPs.

The crystallinity of the NPs was evaluated with a Wide-Angle XRD. Figure 3A shows the XRD patterns of the NPs. All the NPs presented a high amount of an amorphous phase (94–97%), while the total percentage of crystalline phase resulted up to 6%. Specifically, a crystalline phase of Calcite (CaCO_3_) was detected in all the samples, with 6, 4 and 3% for SiCaCe1-NP, SiCaCe2.5-NP and SiCaCe5-NP, respectively. No other calcium or cerium oxides were detected, suggesting the incorporation of the respective ions in the silicate network. Calcium carbonate is a bioactive, biocompatible and biodegradable compound that has attracted considerable interest in regenerative medicine due to its faster resorption and dissolution rates [54,55].

The structure and pore periodicity of NPs were examined via small-angle XRD. As shown in Figure 3B, the spectrum of pure Si–NP has four distinct peaks at 2.38, 4.10, 4.74 and 6.26 2-theta degrees, which is characteristic of the hexagonal tubular pore arrangement in mesoporous silicas. The main peak is also present at the spectra of the rest cation doped NPs, but it is slightly shifted to smaller angles and has a lower intensity, while the smaller peaks have disappeared. This is an indication of the partial disorder of the hexagonal pore arrangement. Based on the (100) peak maximum values (q*) of the spectra, the d-spacing (Bragg spacing = 2π/q*) and the unit call parameter a (a = 2d100/√3 for p6mm structure) are calculated. The results are shown in Table 4.

#### 3.1.5. N_2_ Porosimetry

**Figure 4:**
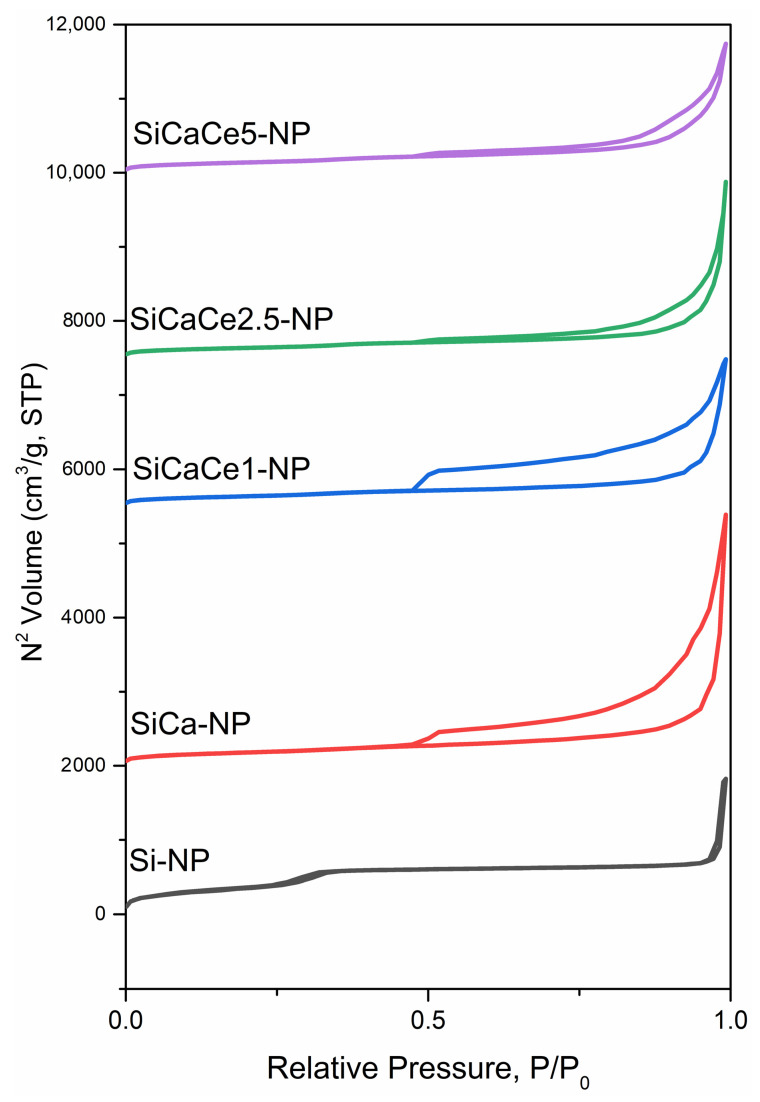
N_2_ adsorption-desorption isotherms of MSNs.

The N_2_ absorption/adsorption isotherms are presented in Figure 4. Pure Si-NP has a Type IV(b) isotherm according to the IUPAC classification, which is characteristic of mesoporous materials. As observed, there are three sharp increases in the curve slope at the following relative pressure P/P0 values: (i) <0.05, (ii) 0.2–0.4 and (iii) >0.9, that correspond to (i) micropore filling, (ii) capillary condensation inside the mesopores and (iii) condensation within the interparticle voids, respectively. Furthermore, there is no hysteresis in the adsorption isotherm, indicating that the diameter of the tubular mesopores is lower than 4 nm. As it concerns the doped NPs, their isotherms are of Type II and the adsorption loop hysteresis is an intermediate of Type H3 and H4. Generally, the Type II isotherm corresponds to non-porous or macroporous materials, but on this occasion, pore blocking by formed CaO might be possible. The H3 Type of hysteresis loop corresponds to layered or macroporous particles with semi-filled pores, while the H4 hysteresis loop is usually found in mesoporous materials with Type I or II isotherms. Finally, as can be seen in the results of Table 1, as the number of different doping ions increases, the specific surface area of the samples decreases, while the pore diameter increases. It is also noteworthy that the doping with various quantities of Ce, up to 0.6/0.05 TEOS/Ce ratio, did not affect the specific area or the pore diameter of the NPs.

#### 3.1.6. Apatite Forming Ability

**Figure 5:**
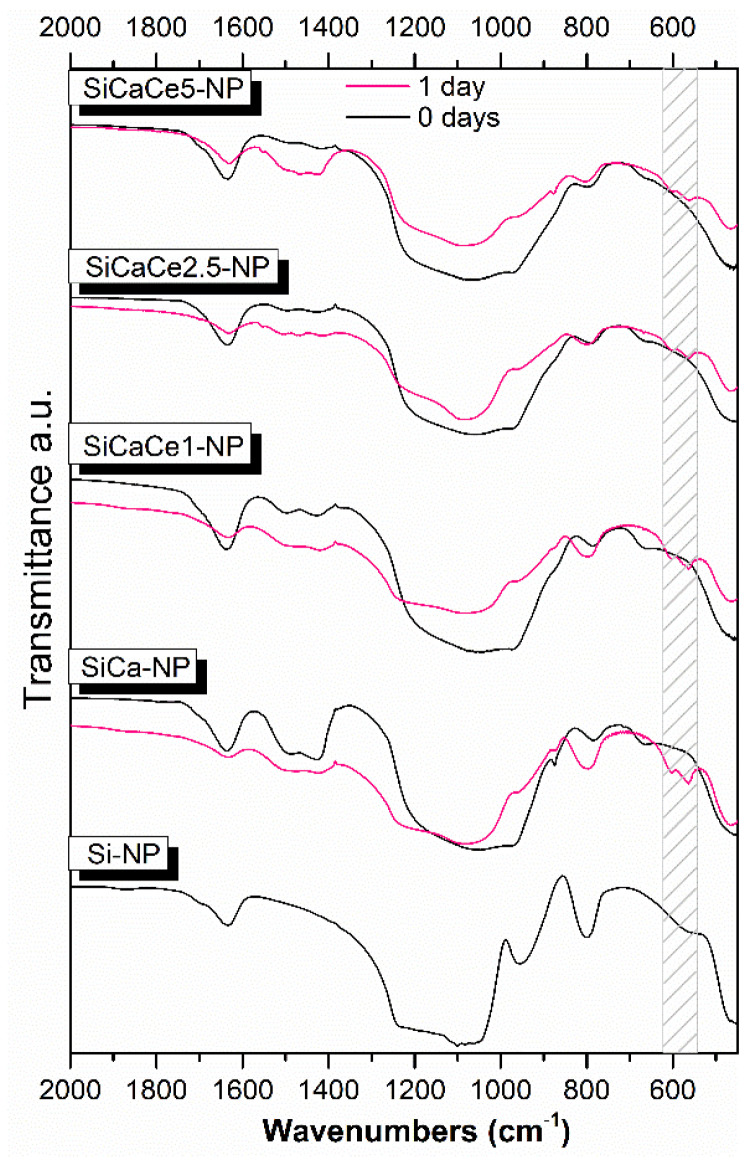
FTIR spectra of all NPs before (0 days) and after (1 day) immersion in simulated body fluid (c-SBF).

Figure 5 shows the FTIR spectra of all the NPs after soaking in simulated body fluid (c-SBF) for 24 h. The FTIR spectra of the different NPs did not show remarkable differences. The spectra of all samples revealed the formation of apatite, due to the sharpening of the broad peak at 900–1200 cm^−1^ attributed to the bending of the (PO4)^3−^ group and the appearance of the double peak at 610–600 and 580–550 cm^−1^ assigned to the P–O bending vibration of HAp [56,57,58]. Additionally, the sharpening of the peak around 780–800 cm^−1^ corresponds to the Si–O–Si stretching vibration due to the polycondensation step of silanols [58,59,60].

Highly bioactive glasses are able to elicit a biological response at their surface that leads to the formation of a bond with the tissues when they are soaked in human plasma [61]. As mentioned before, there is a relation between the percentage and kind of Si–O–NBO of the pristine material and the formation of silica-gel and Ca-P layers [31]. Bioactive glasses containing a high amount of NBO in the glass network present favorable ion exchange conditions, important for the formation of silanol (Si–OH) groups, the subsequent condensation of the SiO_2_ layer and the formation HCAp on their surface [31].

### 3.2. Artemisinin Loading and Release

**Figure 6:**
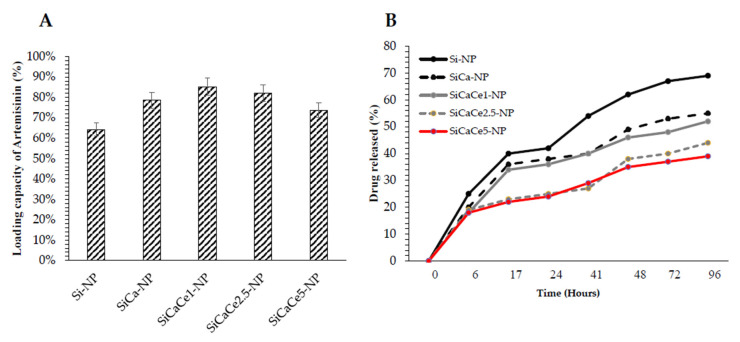
(**A**) Loading Capacity of Artemisinin in different groups of mesoporous NPs, (**B**) Artemisinin release capacity (%) of mesoporous NPs in a time range of up to 96 h.

Figure 6A summarizes the results of the drug loading capacity for each sample. The rates varied from 64.2 to 85.2%, indicating a medium to high loading capacity, whereas the Ca-doped and Ce-doped NPs exhibited higher drug content than that of pure silica (Si-NP), revealing that the addition of Ca and Ce in the system enhances the loading capacity.

In Figure 6B, the release capacity of the ART-loaded NPs up to 96 h is presented. An initial burst release was recorded for all the NPs, while all the Ce-doped NPs, especially those with the higher doping, presented a more sustained release until the first 48 h. After that, a slow-going release followed, eventually reaching almost a plateau, suggesting the formation of a strong chemical affinity between the Ce-doped NPs and ART, which, however, needs further investigation. The SiCaCe2.5-NP presented a loading capacity over 80%, the second highest loading capacity after SiCaCe1-NP, with the most sustained release, similar to SiCaCe5-NP. Although the differences in loading capacity were not remarkable among the Ce-doped nanopowders, the most promising results come from the SiCaCe2.5-NP. This may be attributed to the possible differences in the ratio of Ce^3+^/Ce^4+^ that may have slightly affected the binding efficacy to ART, although this assumption needs further investigation. 

### 3.3. Biological Behavior of NPs and ART-Loaded NPs

#### 3.3.1. Hemocompatibility Assay

**Figure 7:**
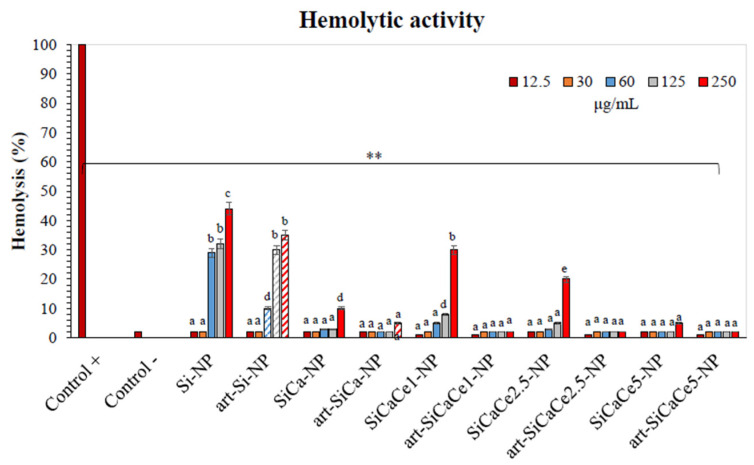
Hemocompatibility assay of NPs and ART-loaded NPs after 60 min of incubation at 37 °C). ** indicates statistically significant difference (*p* < 0.001) between treated cells and untreated (controls), while different letters suggest statistically significant differences (*p* < 0.001) among concentrations.

A similar trend was observed for the evaluated times of incubation (from 3 min to 24 h), indicating a stable interaction of NPs with the red blood cells (RBCs). Figure 7 presents the hemolytic activity of NPs after 60 min of incubation at body temperature (37 °C). All the NPs did not induce hemolysis in concentrations lower than 30 μg/mL. Undoped Si-NP presented hemolysis starting from 60 μg/mL, but doped NPs presented better hemocompatibility at higher concentrations (125 μg/mL for Ce-NPs and 250 μg/mL for Ca-NP). The average Ce-NPs concentration to induce 5% hemolysis was 60 μg/mL. SiCaCe5-NP did not show hemolytic activity and, in addition, presented the best hemolytic compatibility in comparison with all the tested doped materials (*p* < 0.01), reinforcing the view that cerium in the right proportion is blood compatible. Ion doping with cerium in the investigated calcium silicate system induces a protective effect in an erythrocyte membrane in comparison with pure silica. The doping of silica nanoparticles with various ions such as Ca, Mg and Cu can improve their performance and further improve the interaction with human cells [31,62]. Regarding the artemisinin-loaded NPs, all the tested concentrations of the ion-doped loaded NPs appeared with a 100% hemocompatibility, indicating the beneficial role of artemisinin in porous materials, increasing their properties of compatibility in terms of cell viability. On the other hand, the undoped art-loaded NPs presented hemolysis in concentrations higher than 60 μg/mL but with lower percentages of lysis in comparison with the unloaded NPs. Artemisinin and artemisinin derivatives loaded nanoparticles have shown reduced or no hemolytic activity, suggesting a potential protective effect on RBCs’ lysis [63,64]. However, the literature is limited in this area and future studies should be focused on the protective role of ART on nanoparticles entering blood circulation.

#### 3.3.2. Cytotoxicity Assay

**Figure 8:**
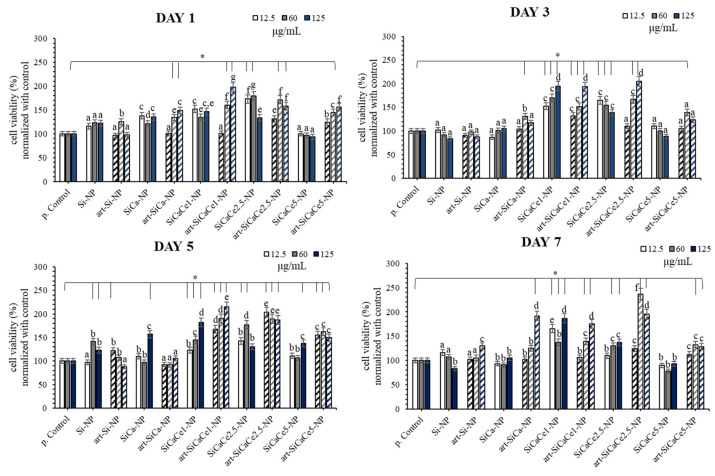
MTT results of cell viability at different concentrations of nanopowders (NPs) (μg/mL). The lines with * above bars indicate statistically significant differences (*p* < 0.05) of cell viability among the treated cells with the different NPs and concentrations and the untreated cells (controls), while different letters above bars suggest statistically significant differences (*p* < 0.05) of cell viability among the cells treated with different concentrations of NPs. Same letters above the bars suggest that cell viability did not differ significantly among the specific NPs and associated concentrations.

The cytotoxicity results after the incubation of nanopowders (NPs) for 1, 3, 5 and 7 days are presented in Figure 8. Statistical analysis revealed significant differences between different groups regarding doping and loading capacity, their dilutions and time of incubation. All the tested undoped, doped and loaded mesoporous NPs presented non cytotoxic behavior and cell viability increased in most of the cases. In detail, at day one, an increase in mitochondrial activity and, consequently, cell viability was observed for all the tested groups compared with the positive control. The most pronounced and statistically significant increase (*p* < 0.05) was reported for the cells treated with art-SiCaCe1, SiCaCe1-NP, art-SiCaCe2.5 and SiCaCe2.5-NP. A dose dependency effect was observed for the cells treated with the art-loaded NPs (*p* < 0.05). Although non cytotoxic biological behavior was confirmed at day three, a statistically significant positive effect on cell viability was observed in the case of SiCaCe-NPs and art-SiCaCe-NPs (*p* < 0.05). Moreover, at day three, a statistically significant dose dependency (*p* < 0.001) was reported for all the Ce-doped NPs and art-loaded NPs (except art-SiCaCe5), indicating 125 μg/mL as the most beneficial concentration concerning cell viability. Enhanced cell proliferation was also observed at day five for all the tested groups and especially for the art-loaded NPs, indicating the capacity of artemisinin to enhance the capability of cells to proliferate. Finally, at day seven, cell proliferation was promoted significantly in the cases of SiCaCe1-NP and all the art-loaded NPs (*p* < 0.05). At this time point, a statistically significant reduction in cell viability was observed only in the case of SiCaCe5-NP (*p* < 0.05). The highest increase in cell viability was reported after treatment with art-loaded SiCaCe1 and -SiCaCe2.5 at all the tested time points, in comparison with the positive control (*p* < 0.05). The unloaded SiCaCe1-NP and SiCaCe2.5-NP groups presented very similar biological behavior with the art-loaded releasing artemisinin for extra enhancement of cell proliferation at all-time points. The art-loaded and unloaded SiCaCe1-NP group presented even superior effect in comparison with SiCaCe2.5-NP after 7 days of cell culture (*p* < 0.05). 

In agreement with the present results, it is supported in the literature that fibroblasts’ biological reaction/behavior appears to be sensitive to lanthanoid stimulation [65]. More specifically, although lanthanoid elements (including Ce) have an ionic radius similar to that of Ca^2+^, they present a higher overall charge density [66]. That is why lanthanoid ions capture Ca^2+^ binding sites on proteins and may have an impact on cells’ proliferation or differentiation. This molecular pathway may provide a possible explanation for the superior biological behavior of Ce-doped mesoporous NPs compared with Ca-doped ones. In the present study, the lower tested concentrations of Ce were the most effective concerning the cells’ proliferation. Zhang et al. [67] supported that higher concentrations of lanthanoid ions could restrict the differentiation of osteoblasts at early time points; however, it could affect their proliferation positively, as these two functions (proliferation/differentiation) are actually inverse and complementary. As human periodontal ligament fibroblasts constitute a mixed cellular population and have shown differentiation potential to various cell lines [68,69], future experiments studying hGFs’ differentiation should follow, in order to investigate whether the reduction in proliferation in the case of SiCaCe5-NP could be attributed to the beginning of differentiation or to a cytotoxicity effect due to the higher cerium concentration. 

Nanoparticles present remarkable reactivity in different environments (aqueous, fetal bovine serum, SBF) due to their high surface energy [70]. Dissolution agglomeration and/or aggregation are some of the reactions that could be observed. Aggregation leads to alteration in the geometry and size of nanoparticles and, consequently, could affect their physicochemical and biological properties [71]. An increase in cell viability due to aggregation has been reported [72,73], while the time point of aggregates’ formation seems to be crucial [74]. Cytotoxicity behavior was reported when aggregates were formed, after endocytosis, inside cells. Otherwise, aggregates could adhere on the cells’ surface promoting final cell growth and cell viability. On the other hand, it has been reported that steric stabilization can be expected in proteins containing biological fluids [75]. For example, gold nanoparticles can be stabilized in the presence of proteins while they are aggregated in PBS [76]. Similarly, SiO_2_ nanoparticles can be aggregated or well-dispersed in PBS with a low and a high protein concentration, respectively [77]. Both the protein amount and the chemical composition of nanoparticles can influence stabilization [75]. The complex cellular environment, even during in vitro cultivation in the presence of nanoparticles, is characterized by variability in the amount and type of electrolytes, as different ionic products can be released during nanoparticles’ degradation and differences on culture media components exist, while protein corona formation alters surface charge [78], having a significant effect on nanoparticles’ stability. Regarding the effect of serum in culture media, it has been reported that FBS may facilitate stability [79], while in the presence of FCS, an increased stability of the functionalized nanoparticles was observed [80]. This can be explained by the surface adsorption of the proteins that induce electrostatic repulsion, minimizing aggregation. The effect of aggregation on cell proliferation and growth has not been identified completely and controversial results have been reported. The exact role of the cell type and the size of aggregates needs to be established. As in this in vitro study, the aggregation in cell culture medium (size and shape of aggregates) was not investigated, future investigation will be focused on the identification of these phenomena and the role of aggregates on the NPs’ biologic behavior.

Our results provide a novel insight on the role of artemisinin to promote cell proliferation after loading on cerium-doped mesoporous NPs. In agreement with previous results regarding the advantageous role of artemisinin in dental pulp stem cells (DPSC), bone marrow-derived mesenchymal stem cells (BMSCs) and in human mesenchymal stem cells (hMSCs), we have shown that human periodontal ligament fibroblasts are also positively affected by artemisinin. Ni et al. demonstrated not only the beneficial role of artemisinin but also the possible implication of artemisinin through the signaling pathways of ERK1/2 as well as Wnt/β-catenin to promote osteogenesis in hMSCs. Moreover, in a recent study of Hu et al. artemisinin in dental pulp stem cells reversed the suppression in cell survival affected from hypoxia and was also able to reduce the apoptotic rates and the expressions of pro-apoptotic proteins. Consistent with these findings, our study confirms the significant induction of cell proliferation of cerium doping and artemisinin release in hPDLFs. Very recently, Ren et al. [81] introduced CeO_2_ nanoparticles to promote bone regeneration, which is crucial for complete periodontal tissue regeneration, and reported an increased proliferation and osteogenic differentiation of human periodontal ligament stem cells in contact with the nanoparticles. They justified the upregulation of human periodontal ligament stem cells’ proliferation by the ROS scavenging properties of CeO_2_ nanoparticles, highlighting the need for more research on the role of ceria in the osteogenic differentiation of periodontal ligament cells.

#### 3.3.3. ROS Analysis of hPDLFs after Interaction with NPs

**Figure 9:**
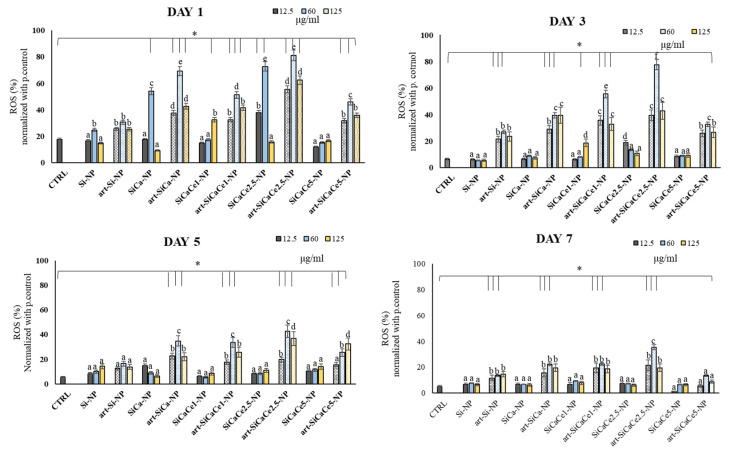
Extracellular ROS (%) levels after 1, 3, 5 and 7 days of different concentrations (12.5, 60, 125 μg/mL)) of NPs in contact with hPDLFs. The lines with * above bars indicate statistically significant differences (*p* < 0.05) of ROS % among the treated cells with the different NPs and concentrations and the untreated cells (controls), while different letters above bars suggest statistically significant differences (*p* < 0.05) of ROS % among the cells treated with different concentrations of NPs. Same letters above the bars suggest that ROS production did not differ significantly among the specific NPs and associated concentrations.

Figure 9 presents the extracellular ROS levels of hPDLFs incubated with various NPs (Day 1, 3, 5 and 7). At day one, an overproduction of free radicals was observed after the exposure of NPs to hPDLFs, compared with the other time points. Furthermore, a statistically significant increase in ROS production was observed for Ce-NPs, especially for SiCaCe1 and SiCaCe2.5 NPs (*p* < 0.05). In detail, SiCaCe1-NP and SiCaCe2.5-NP showed the highest increase in free radical production at 60 μg/mL, 60 and 85%, respectively, compared with the control. The highest tested concentrations allowed a moderate ROS production, while the most significant increase was observed using 60 μg/mL, indicating the crucial role of NPs’ concentrations to encounter ROS related pathways. In all the cases except SiCa1, a time dependency was observed too (*p* < 0.05). At day five and seven, the ROS levels decreased significantly and returned to the normal amounts (basal production of ROS).

Periodontitis is an inflammatory process causing alveolar bone loss, periodontal pocket formation and a generalized destruction of the periodontal apparatus, eventually leading to tooth loss [82]. Although periodontal pathogens are crucial for the establishment of the disease, their interaction with the host immune response determines its progression. Hajishengallis [83] reported that the progress of periodontitis involves interactions among leukocytes, complement and reactive oxygen species (ROS) and recent comprehensive reviews have acknowledged the close relationship between ROS production and periodontitis [84,85]. ROS constitute a family of different highly reactive products of oxygen, such as hydrogen peroxide (H_2_O_2_), superoxide anion (O_2_^−^) and the hydroxyl radical (•OH) [86]. Although ROS are implicated in the normal cellular metabolism and are continuously generated by cells, oxidative stress can be exerted due to an imbalance between ROS production and antioxidants, leading to periodontal tissue destruction through protein and DNA damage, lipid peroxidation and the oxidation of important enzymes [87]. Based on the ROS-mediated periodontal inflammation, strategies to minimize ROS generation or to develop ROS scavenging mechanisms are essential to effectively control the host inflammatory response.

In this respect, we evaluated the total ROS production after contact of hPDLFs with the synthesized nanopowders, either loaded or not with artemisinin. A clear reduction in ROS was observed after the first day of incubation where an overproduction was observed, suggesting that the NPs were effective in neutralizing ROS. When in the form of CeO_2_, the Ce atom exists in both the Ce^3+^ and Ce^4+^ states, creating an autoregenerative redox cycle between Ce^3+^ and Ce^4+^ [88,89]. This reversibility allows the storage and release of oxygen and, thus, cerium oxide can exhibit antioxidant properties by inhibiting NF-κB activation and the expression of inflammatory genes [90], regulating the activity of antioxidant enzymes and intracellular GSH levels [90] and enhancing the cellular defense against free radicals [91]. Numerous studies have revealed that the scavenging properties of CeO_2_ nanoparticles are size- [92,93] and pH-dependent [94,95]. In the present study, cerium nitric salts (Ce^3+^) were used for the synthesis of NPs and XRD analysis revealed the presence of CeO_2_ traces. As shown in the XRF analysis, cerium was successfully incorporated in the NPs, being effective in controlling their antioxidant properties.

## 4. Conclusions

The sol-gel technique was successfully utilized for the development of highly bioactive Ce-doped mesoporous nanopowders. Cerium doping positively affected the cell proliferation of human periodontal ligament fibroblasts; however, at the highest cerium concentration, a mild restriction of cell viability was evidenced after 7 days of culture. Mesoporous nanostructured powders presented an hemocompatible character with the potential to be used at concentrations up to 125 μg/mL. Artemisinin was successfully loaded with a capacity reaching up to 80%, substantially improving the materials hemocompatibility and cells viability. All the tested NPs presented ROS scavenging properties and revealed the crucial role of NPs concentrations to encounter ROS related pathways. Based on the results of the present study, SiCaCe2.5-NP presented the best overall performance in respect to the investigated properties and can be considered as a promising candidate in periodontal ligament regeneration applications.

## Figures and Tables

**Table 1:** Reaction stoichiometry for the produced MSNs.

Sample	Molar Ratio of Reactants
Si-NP	1 TEOS/0.13 CTAB/0.4 NaOH/1240 H_2_O
SiCa-NP	0.6 TEOS/0.13 CTAB/0.4 NaOH/1240 H_2_O/0.4 Ca
SiCaCe1-NP	0.6 TEOS/0.13 CTAB/0.4 NaOH/1240 H_2_O/0.39 Ca/0.01 Ce
SiCaCe2.5-NP	0.6 TEOS/0.13 CTAB/0.4 NaOH/1240 H_2_O/0.375 Ca/0.025 Ce
SiCaCe5-NP	0.6 TEOS/0.13 CTAB/0.4 NaOH/1240 H_2_O/0.35 Ca/0.05 Ce

**Table 2:** Reagents for preparation of SBF (pH 7.30–7.40, 2000 mL).

Order	Reagent	Amount
1	NaCl	16.072 g
2	NaHCO_3_	0.704 g
3	KCl	0.450 g
4	K_2_HPO_4_·3H_2_O	0.460 g
5	MgCl_2_·6H_2_O	0.622 g
6	1.0 M-HCl	6 mL
7	CaCl_2_	0.586 g
8	Na_2_SO_4_	0.144 g
9	(CH_2_OH)_3_CNH_2_(TRIS) ^a^	12.126 g

^a^ TRIS: tris(hydroxymethyl)amiomethne.

**Table 3:** Chemical composition of the synthesized MSNs as detected using XRF in mol%.

Sample	SiO_2_		CaO		CeO_2_		Total N	Total XRF
	N	XRF	N	XRF	N	XRF		
Si-NP	100	100	-	-	-	-	100	100
SiCa-NP	61.64	61.48	38.36	38.52	-	-	100	100
SiCaCe1-NP	60.44	70.74	36.67	27.92	2.89	1.34	100	100
SiCaCe2.5-NP	58.73	72.13	34.26	23.19	7.01	4.68	100	100
SiCaCe5-NP	56.08	71.84	30.53	19.64	13.39	8.52	100	100

N = Nominal composition.

**Table 4:** Structural and textural properties of NPs.

Sample	XRD		N_2_ Porosimetry		
	d-Spacing	*α*_0_	*S*_BET_	*d*_p_	*V*_p_
	(nm)	(nm)	(m^2^/g)	(nm)	(cc/g)
Si-NP	3.71	12.85	1312	2.5	2.82
SiCa-NP	4.96	17.18	650	4.1	5.24
SiCaCe1-NP	5.07	17.57	495	3.9	3.07
SiCaCe2.5-NP	5.32	18.42	495	3.9	3.68
SiCaCe5-NP	5.52	19.11	495	3.9	2.69

## Data Availability

Data are contained within the article.
